# Dynamic BAF chromatin remodeling complex subunit inclusion promotes temporally distinct gene expression programs in cardiogenesis

**DOI:** 10.1242/dev.174086

**Published:** 2019-07-05

**Authors:** Swetansu K. Hota, Jeffrey R. Johnson, Erik Verschueren, Reuben Thomas, Aaron M. Blotnick, Yiwen Zhu, Xin Sun, Len A. Pennacchio, Nevan J. Krogan, Benoit G. Bruneau

**Affiliations:** 1 Gladstone Institutes, San Francisco, CA 94158, USA; 2 Roddenberry Center for Stem Cell Biology and Medicine at Gladstone, San Francisco, CA 94158, USA; 3 Department of Cellular and Molecular Pharmacology, University of California San Francisco, San Francisco, CA 94158, USA; 4 Genomics Division, Lawrence Berkeley National Laboratory, Berkeley, CA 94720, USA; 5 United States Department of Energy, Joint Genome Institute, Walnut Creek, CA 94598, USA; 6 Department of Physiology, Anatomy and Genetics, University of Oxford, Oxford OX1 3PT, UK; 7 Department of Pediatrics, University of California, San Francisco, CA 94143, USA; 8 Cardiovascular Research Institute, University of California, San Francisco, CA 94158, USA

**Keywords:** Heart, Differentiation, Chromatin, Gene regulation

## Abstract

Chromatin remodeling complexes instruct cellular differentiation and lineage specific transcription. The BRG1/BRM-associated factor (BAF) complexes are important for several aspects of differentiation. We show that the catalytic subunit gene *Brg1* has a specific role in cardiac precursors (CPs) to initiate cardiac gene expression programs and repress non-cardiac expression. Using immunopurification with mass spectrometry, we have determined the dynamic composition of BAF complexes during mammalian cardiac differentiation, identifying several cell-type specific subunits. We focused on the CP- and cardiomyocyte (CM)-enriched subunits BAF60c (SMARCD3) and BAF170 (SMARCC2). *Baf60c* and *Baf170* co-regulate gene expression with *Brg1* in CPs, and in CMs their loss results in broadly deregulated cardiac gene expression. BRG1, BAF60c and BAF170 modulate chromatin accessibility, to promote accessibility at activated genes while closing chromatin at repressed genes. BAF60c and BAF170 are required for proper BAF complex composition, and BAF170 loss leads to retention of BRG1 at CP-specific sites. Thus, dynamic interdependent BAF complex subunit assembly modulates chromatin states and thereby participates in directing temporal gene expression programs in cardiogenesis.

## Abstract (precis)

**Summary:** BRG1/BRM-associated factor (BAF) chromatin remodeling complexes include specific subunits during cardiac differentiation that are important for BAF complex composition and for regulating cardiac gene expression.

## INRODUCTION

Cell differentiation and organogenesis are regulated by the precise transcriptional output of a coordinated gene regulatory network (Davidson, 2010). During mammalian development, gene expression programs are spatially and temporally controlled, with specific sets of genes being expressed while others are poised or repressed in a developmental stage-dependent manner. It is considered that differentiation proceeds as a gradually increasing specification of cell fates (Moris et al., 2016). Delineating the factors that control crucial developmental decision points is essential to understand the control of gene regulation during differentiation and development.

Transcription factor (TF) activity regulates transcriptional output, and is intimately influenced by the underlying chromatin. Chromatin remodeling complexes are multi-subunit protein complexes that alter histone-DNA contact in nucleosomes to reorganize chromatin and regulate transcription (Bartholomew, 2014; Clapier and Cairns, 2009; Zhou et al., 2016). The BRG1/BRM-associated factor (BAF) chromatin remodeling complexes are composed of the mutually exclusive brahma (BRM) or brahma-related gene 1 (BRG1; also known as SMARCA4) ATPAses, along with several other structural subunits and their isoforms. These form diverse BAF complexes that serve specific functions in widely different cell types and developmental processes (Ho and Crabtree, 2010; Hota and Bruneau, 2016; Wang et al., 1996). BAF complexes have a specific composition in certain cell types, e.g. an embryonic stem cell complex that regulates pluripotency (Ho et al., 2011, 2009b) or a neural precursor and neural BAF complexes that fine-tune neurogenesis (Lessard et al., 2007; Staahl et al., 2013). Current evidence indicates that a shift in isoforms of non-essential BAF complex subunits is important for the stepwise transition from a precursor to a differentiated state in neural and muscle differentiation (Goljanek-Whysall et al., 2014; Lessard et al., 2007; Saccone et al., 2014; Staahl et al., 2013; Wu et al., 2007). Mutations in genes encoding BAF complex subunits have been associated with various cancers (Pierre and Kadoch, 2017), and some have also been found in cases of congenital heart disease (CHDs) (Jin et al., 2017). In the developing heart, subunits of the BAF complex are involved in diverse aspects of cardiac development (Chang and Bruneau, 2012; Sun et al., 2018). *Brg1* (*Smarca4*) is haploinsufficient in the mouse heart, and genetically interacts with genes encoding DNA-binding transcription factors associated with CHDs, indicating a potentially general role for BAF complexes in these common birth defects (Takeuchi et al., 2011).

Differentiation is thought to proceed during development as a continuous but highly regulated series of milestones, which include lineage decisions and linear progression towards a terminally differentiated state. These sequential events can be modeled effectively using pluripotent stem cells that are subjected to well-defined differentiation protocols (Loh et al., 2016; Murry and Keller, 2008). Cardiac differentiation is composed of a stereotyped set of steps, with the initial formation of cardiogenic mesoderm, subsequent specialization into multipotent cardiac precursors (CPs) and then differentiation into beating cardiomyocytes (CMs) (Devine et al., 2014; Evans et al., 2010; Kattman et al., 2011; Wamstad et al., 2012). The *in vivo* embryonic steps are well recapitulated in *in vitro* differentiation protocols (Kattman et al., 2011; Wamstad et al., 2012). It is not known which BAF complex subunits are essential for controlling temporal steps in cardiac differentiation.

Here, we define dynamic BAF complex composition during mouse CM differentiation. We identify several BAF complex subunits as enriched in CPs and CMs, and along with BRG1 chose to focus on BAF60c (also known as SMARCD3) and BAF170 (also known as SMARCC2). BAF60c is expressed preferentially in the developing heart, and is important for its morphogenesis (Lickert et al., 2004; Sun et al., 2018). BAF60c and BAF170 together are the first subunits to assemble during BAF complex formation (Mashtalir et al., 2018) and thus may have shared functions. We find that BRG1 initiates cardiac gene expression programs in precursor cells, a role shared by BAF60c and BAF170, which also maintain the cardiac program to facilitate CM differentiation. BAF60c and BAF170 also regulate BAF complex composition, and their loss alters chromatin accessibility. Furthermore, we find that BAF170 facilitates dissociation of BRG1 complexes from their binding sites upon CM differentiation. These results reveal the instructive nature that a specific combination of BAF subunits attains to dictate functional outcomes during lineage commitment and differentiation.

## RESULTS

### Brg1 initiates cardiac gene expression programs during CM differentiation

**Fig. 1.**
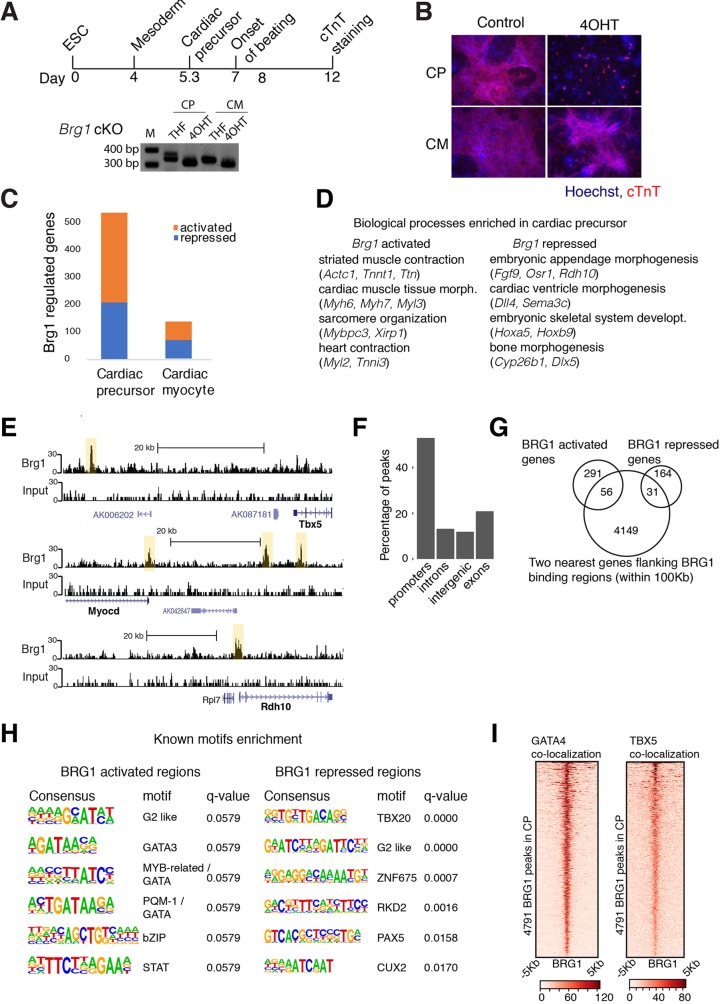
**BRG1 directs cardiac gene expression.** (A) Schematics of cardiac differentiation. 4-hydroxyl tamoxifen (4OHT) was added at D4 and D8 to conditionally delete *Brg1* at CP and CM, respectively. Genotyping PCR shows a *loxP* site containing a 350 bp band and a faster migrating 313 bp band after addition of 4-OHT. (B) Immunofluorescence of cTnT at day 10 of differentiation. (C) RNAseq of BRG1-regulated genes at CP and CM stages. (D) Gene ontology (GO) biological processes enriched in BRG1 activated and repressed genes in CPs. (E) Input and BRG1 ChIPseq browser tracks in CPs. (F) Classification of BRG1 ChIPseq peaks into different genomic regions. Promoters are defined as being within 1 kb of the transcription start site. (G) BRG1-activated or -repressed genes overlapping with the two nearest genes flanking the BRG1-binding sites shown in a Venn diagram. (H) Motifs enriched in BRG1-binding sites associated with BRG1-activated and -repressed genes. (I) GATA4 and TBX5 binding correlates well over BRG1-binding sites in CPs.

To model early heart development, we differentiated mouse embryonic stem cells (mESCs) to cardiac troponin T-positive (cTnT^+^) beating cardiac myocytes (Kattman et al., 2011; Wamstad et al., 2012). To understand the role of the BAF complex ATPase BRG1 during CM differentiation, we conditionally deleted *Brg1* using an inducible Cre-loxP system (Alexander et al., 2015; Ho et al., 2009b). Addition of 4-hydroxy tamoxifen effectively deleted exon 17 and 18 of the *Brg1* gene encoding part of ATPase domain (Fig. 1A), leading to a greater than 90% reduction in BRG1 protein within 36 h (Alexander et al., 2015). Loss of *Brg1* at the CP stage, but not in CMs, inhibited CM differentiation (Fig. 1B). RNAseq revealed that *Brg1* regulated a total of 545 genes in CPs and 125 genes in CMs (*P*<0.05, ±1.5-fold). Reduced importance of *Brg1* at the CM stage is consistent with its reduced expression (Fig. 1C and Fig. S1A). In CPs, *Brg1* repressed 197 (36.1%) and activated 348 (63.9%) genes (Fig. 1C and Table S4). BRG1-activated genes were enriched for sarcomere organization and assembly, and are essential components of cardiac cell fate establishment (Fig. 1D). Of note, *Brg1* is essential for the activation of these lineage-specific genes, in anticipation of the final differentiation status of the cells, but not for their maintenance. Thus, *Brg1* primes the cell type-specific differentiation of CPs. In CMs, although BRG1 activated and repressed roughly equal number of genes, it did not significantly enrich for any biological processes, consistent with *in vivo* data (Hang et al., 2010).

### BRG1 binding correlates with sites of cardiac transcription factor binding

**Fig. 2.**
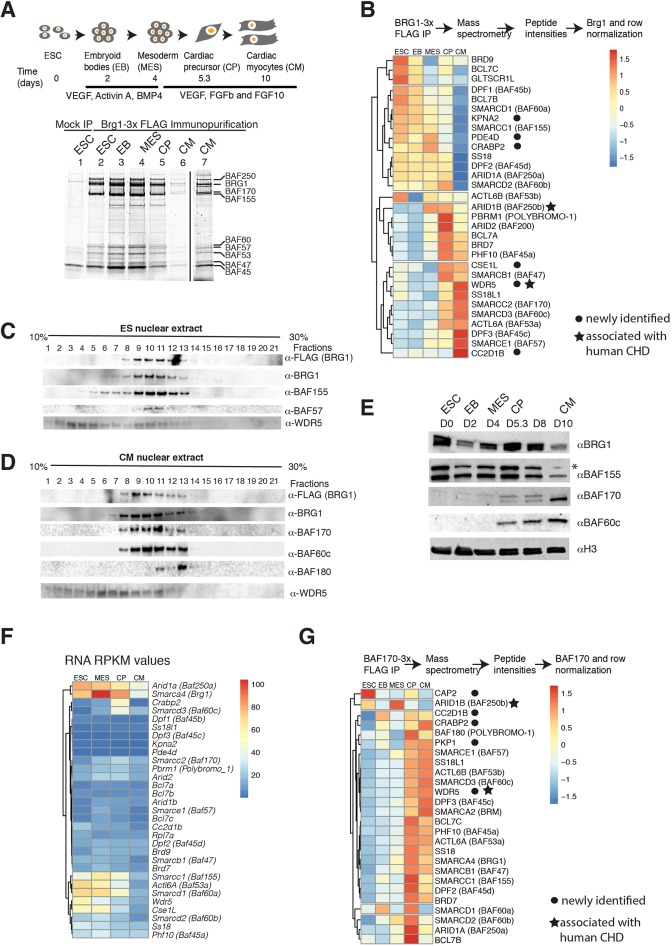
**BRG1-containing complexes dynamically change subunit composition during cardiac differentiation.** (A) Schematic representation of cardiac differentiation and isolation of BRG1 complexes by anti-FLAG immunopurification. SDS-PAGE showing BRG1 complexes from an untagged (lane 1) and a Brg1-3x FLAG tagged mESC line differentiated to CMs at different stages (lanes 2-6). The BRG1 complex isolated at the CM stage (lane 6) is shown with increased contrast in lane 7. Protein complexes were analyzed using 10% SDS-PAGE and stained with SyproRuby protein gel stain. Protein labels are based on molecular mass and the nomenclature of BRG1-associated factors (BAFxx, where xx is molecular mass). These bands were not individually excised and identified. (B) Mass spectrometric analysis of BRG1 complexes. The peptide intensities are normalized to mock untagged intensities and to BRG1 intensities, and are further normalized across the row. Blue and red indicate depletion and enrichment, respectively; yellow indicates unchanged. Nuclear extracts of ESCs (C) or CMs (D) resolved in a 10-30% glycerol gradient and BRG1-associated subunits detected by western blot. (E) Nuclear extracts resolved in 10% SDS-PAGE from different stages of cardiac differentiation detected BAF/PBAF subunits by western blot. (F) A RNAseq expression profile (RPKM) of BAF/PBAF subunits during cardiac differentiation. (G) Peptide intensities of immunopurified BAF170-3×FLAG protein after immunopurification-mass spectrometry, normalized to mock and to BAF170 proteins, and across stages of differentiation. Blue and red indicate depletion and enrichment, respectively; yellow indicates unchanged.

To understand where BRG1 binds in the genome during cardiac differentiation, we performed ChIPseq using a native antibody against BRG1. In CPs, we found 4791 significant BRG1-binding regions (Fig. 1E and Fig. S1B) and could detect weaker BRG1 occupancy in CMs (Fig. S1C), presumably owing to the low levels of protein at this stage (Fig. 2A,E). In CPs, BRG1 was bound to both transcriptional start sites (TSS) and H3K27ac marked active enhancer regions (Wamstad et al., 2012) (Fig. 1F and Fig. S1D) and associated with 5274 genes (the two nearest genes flanking a BRG1-binding site within 1 Mb). 21.9% of BRG1-activated and 20.9% of the BRG1-repressed genes overlapped with genes nearest to a BRG1-binding site. Putative BRG1-activated targets were enriched for cardiac muscle development, contraction and circulatory system processes, whereas putative BRG1-repressed targets were enriched for embryonic limb and skeletal system development (Fig. S1E). Similar enrichment of biological processes was observed for the two nearest genes flanking the BRG1-binding sites within 100 kb (Fig. 1G and Fig. S1F). These results show that BRG1 facilitates cardiac gene expression programs while preventing other developmental programs.

Motif enrichment analysis using HOMER (Heinz et al., 2010) revealed GATA motifs near BRG1-activated sites and T-box motifs near BRG1 repressed sites, among others (Fig. 1H). Consistently, BRG1-binding sites correlated well with GATA4- and TBX5-binding sites in CPs (Fig. 1I) (Luna-Zurita et al., 2016), suggesting that BRG1 interacts with cardiac TFs to regulate gene expression during cardiac lineage commitment, as predicted from gain-of-function experiments (Lickert et al., 2004; Takeuchi and Bruneau, 2009). Thus, BRG1 (and BRG1 containing complexes), likely in collaboration with cardiac transcription factors, direct the cardiac gene expression program while preventing expression of non-cardiac genes.

### The BRG1 complex shows dynamic composition during CM differentiation

To understand the composition of BRG1-associated complexes during cardiac differentiation, we immunopurified BRG1 using a mouse embryonic stem (ES) cell line in which a 3×FLAG epitope tag was added to the endogenous BRG1 open reading frame (Attanasio et al., 2014), under moderately stringent binding and wash conditions (300 mM NaCl, 0.02% NP40), and competitively eluted BRG1-containing complexes in presence of 0.1 mg/ml 3×FLAG peptide. We isolated BRG1 complexes from five different stages of cardiac differentiation: ES cells (ESCs), embryoid bodies (EBs), cardiac mesoderm (MES), cardiac progenitor (CP) and cardiac myocytes (CM) in biological triplicates (Fig. 2A). An untagged control cell line similarly processed in biological triplicates at each stage served as a negative control. BRG1 complexes isolated from each of these stages showed protein profiles similar to previously reported complexes, and we have annotated the bands based on these well-described complex migration patterns, keeping in mind that each band was not excised and identified (Fig. 2A) (Ho et al., 2009b). We performed mass spectrometry (MS) in biological triplicates and technical duplicates, and compared peptide intensities after normalizing against an untagged control, the bait BRG1 protein, and across stages of differentiation, and identified the composition of the BRG1 complexes at these five stages of cardiac differentiation (Fig. 2B, Tables S1-S3). This resulted in highly statistically significant identification of BAF complex subunits. ESC-derived BRG1 complexes were enriched for BRD9, GLTSCR1l (BICRAL), BCL7b, BCL7c, BAF45b (DPF1), BAF155 (SMARCC1) and BAF60a (SMARCD1), consistent with previous reports (Ho et al., 2009b; Kadoch and Crabtree, 2013). We identified proteins enriched at mesoderm (PDE4D, CRABP2, ARID1B), CP [BAF60b (SMARCD2), POLYBROMO-1 (PRBM1), ARID2, BAF47 (SMARCB1), BCL7a, BRD7 and BAF45a) and CM stages [BAF170 (SMARCC2), BAF60c (SMARCD3), BAF57 (SMARCE1), SS18l1, BAF45c (DPF3), WDR5 and CC2D1B]. New BRG1-interacting factors were identified, including CRABP2, KPNA2, PDE4D, CSE1L, CC2D1B and, intriguingly, WDR5. WDR5 is well known to be part of the MLL complex, and has been implicated in human CHD (Zaidi et al., 2013). Glycerol gradient experiments showed co-sedimentation of BAF/PBAF subunits in ES cells and CMs, including WDR5 (Fig. 2C,D). Notably, we did not identify cardiac transcription factors that we previously suggested associate with BAF complexes (Lickert et al., 2004; Takeuchi and Bruneau, 2009; Takeuchi et al., 2011), likely owing to transient and weak interactions with the complex. Functional assessment of BRG1 complexes by *in vitro* nucleosome repositioning or ATPase activity assays indicated that stage-specific complexes had similar activities (Fig. S2A-C). Thus, BRG1 complexes have dynamic subunit composition that gradually changes during cardiac differentiation and is enriched for specific subunits in cardiac lineages.

These results suggest that the BRG1 complex changes its composition during cardiac differentiation and BAF subunits switch from one isoform to other (e.g. BAF60a in ES is replaced by BAF60c in CP/CM BAF complexes) or to a different protein (BAF155 in ES cells to BAF170 in cardiac cells). The switch from BAF155 in ES BAF to more abundant BAF170 in CP/CM, and the appearance of BAF60c only in cardiac cell lineages during differentiation were consistent with their expression pattern (Fig. 2E). However, most subunits were not transcriptionally regulated, and thus the assembly reflects developmental stage-specific inclusion of these subunits (Fig. 2F).

To understand further the composition of cardiac-enriched complexes, we immunopurified BAF170 using a mouse ESC line in which a 3×FLAG epitope tag was added to the endogenous BAF170 open reading frame at five different stages of cardiac differentiation in biological triplicates. A non-FLAG-tagged line processed similarly at each stage served as a negative control. We eluted protein with FLAG peptides and analyzed FLAG purified material by mass spectrometry. Many of the BRG1-associated proteins at the CP and CM stages using BRG1 as bait were also enriched using BAF170 as bait, with certain exceptions (Fig. 2G). For example, ARID1b is enriched in the BRG1 immunopurification-MS at the MES-CM stages, whereas in the BAF170 immunopurification-MS it is depleted. WDR5 was also present in the complexes isolated by BAF170-immunopurification, supporting its association with BAF complexes. In addition, we detected the alternate ATPase BRM in the BAF170-FLAG purification, indicating that BAF170 functions within separate BRG1 and BRM complexes. These results suggest that dynamic subunit composition and subunit switch are important aspects of BAF complexes during cardiac differentiation.

### BAF170 and BAF60c facilitate CM differentiation

**Fig. 3.**
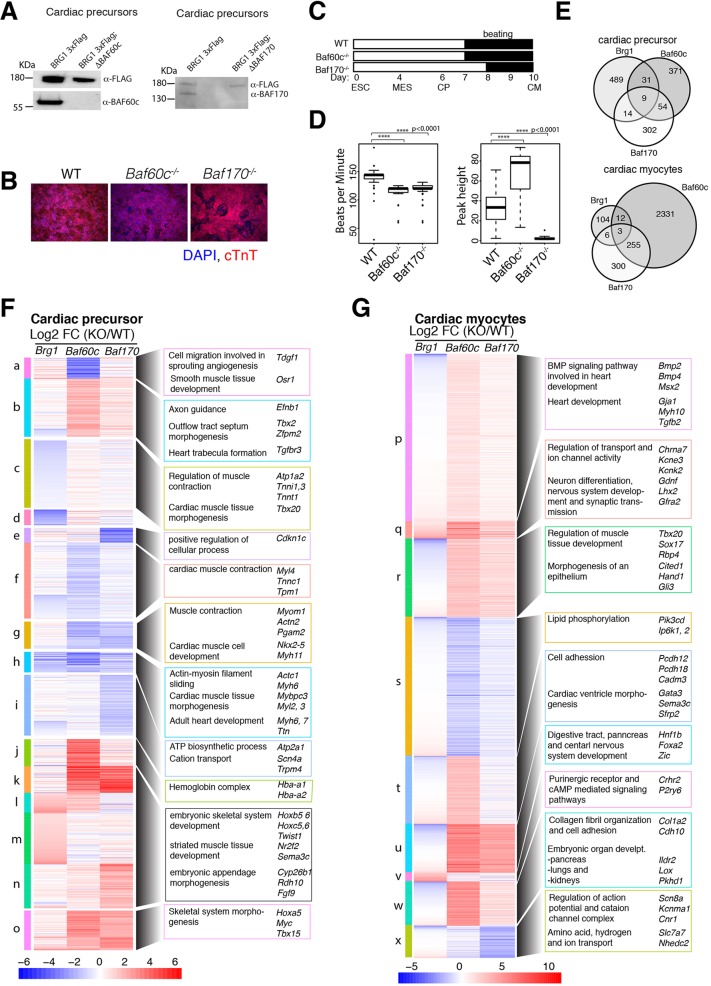
**BAF60c and BAF170 regulate both common and distinct gene expression programs compared with BRG1.** (A) Loss of BAF60c and BAF170 proteins in respective KO cells detected by western bot in CPs. BRG1 levels are shown as loading controls. (B) Immunofluorescence of cardiac troponin T protein in wild-type, BAF60cKO and BAF170 KO cells in CMs. (C) Schematics showing the onset of beating in differentiating CMs (an average of multiple wells and differentiations). (D) Assessments of beating properties of wild-type, BAF60c KO and BAF170 KO CMs obtained by measuring the number of beats per minute and amplitude of beating represented by peak heights. Boxplots show data from the 1st quartile to the 3rd quartile with horizontal lines indicating the median. Whiskers are 1.5 times the interquartile range, with outliers shown outside of the whiskers. (E) Venn diagram shows an overlap of significantly affected genes (±1.5-fold, *P*<0.05) in the absence of *Brg1*, *Baf60c* and *Baf170* at CP and CM stages. (F,G) Heat maps comparing genes significantly deregulated in any of the three genotypes over their respective wild-type counterpart at CP (F) and CM (G) stages. Clusters are shown in vertical colored bars on the left and representative genes involved in various biological processes are shown on the right of the heat map.

Having established the dynamic composition of BAF complexes in cardiac differentiation, we selected two subunits for functional studies: BAF60c and BAF170. BAF60c is known to be important for cardiac differentiation and morphogenesis *in vivo*, although little is known about BAF170 in this context. We investigated the functional roles of *Baf60c* and *Baf170* by deleting them in ES cells using TALEN or CRISPR strategies (Fig. 3A). Both *Baf60c* KO and *Baf170* KO cells underwent cardiac differentiation as observed by beating cardiac myocytes and immunostaining of cardiac Troponin T (Fig. 3B). However, cells lacking BAF170 had a delay in the onset of beating (Fig. 3C) and both *Baf60c* and *Baf170* KO cells displayed significant aberrations in beating, including number of beats per minute and amplitude of beating (peak heights) (Fig. 3D), which are indicative of abnormal cardiac differentiation. Directed differentiation phenotypes must, of course, be interpreted with caution, as a defective gene regulation program may be overcome by a strong set of instructive signals, resulting in a superficially perceived mild or absent phenotype. For example, cells lacking both the cardiac transcription factors NKX2-5 and TBX5 undergo cardiac differentiation but with alterations in gene expression (Luna-Zurita et al., 2016). Mice lacking BAF60c have impaired cardiac morphogenesis, clearly indicating a crucial role for this subunit, but do have CMs, consistent with our *in vitro* observations (Sun et al., 2018). Nonetheless, to understand how these subunits regulate gene expression, we collected cells at CP and CM stages, and carried out RNAseq. In *Baf60c* KO CPs, a total of 474 genes (*P*<0.05, ±1.5-fold) were deregulated, of which 45% were activated and 55% were repressed, while a total of 382 genes are deregulated in *Baf170* KO CPs, of which roughly equal number of genes were up- or downregulated (Fig. S3A, Tables S5 and S6). Gene ontology analyses revealed that *Brg1*- and *Baf60c*-dependent genes share common functions in activating genes involved in cardiac and muscle tissue development and contraction, while repressing skeletal system and limb morphogenesis (Fig. S3C). *Baf170*-dependent genes in CPs are involved in skeletal system morphogenesis and pattern specification (Fig. S3C). However, in both *Baf60c* and *Baf170* KO CMs, a large percentage of genes were repressed. In *Baf60c* KO CMs, out of a total 2646 genes, 72% were repressed and 28% were activated, while in *Baf170* KO CMs, out of 574 genes, 63% and 37% were repressed and activated, respectively (Fig. S3B). *Baf60c* KO CM repressed genes were mostly involved in biological adhesion, developmental process and cell motility, whereas *Baf170* KO CM repressed genes are involved in cell differentiation and cellular developmental processes (Fig. S3D). These results indicate a largely repressive function of these cardiac-enriched subunits in gene regulation.

Overall comparison of the gene expression changes in CPs and CMs lacking BRG1, BAF60c or BAF170 show that all three have shared functions in CP gene expression, but that in CPs and CMs, BAF60c and BAF170 regulate a cohort of genes independently of BRG1 (Fig. 3E-G). These results could be due to an accrued effect of BAF60c or BAF170 loss in gene expression during cardiac differentiation or divergent role of these two subunits from BRG1, suggesting their association in a different BAF complex or potential independent function outside of BAF complexes.

### BAF subunits modulate temporal chromatin accessibility and facilitate cardiac gene expression programs

**Fig. 4.**
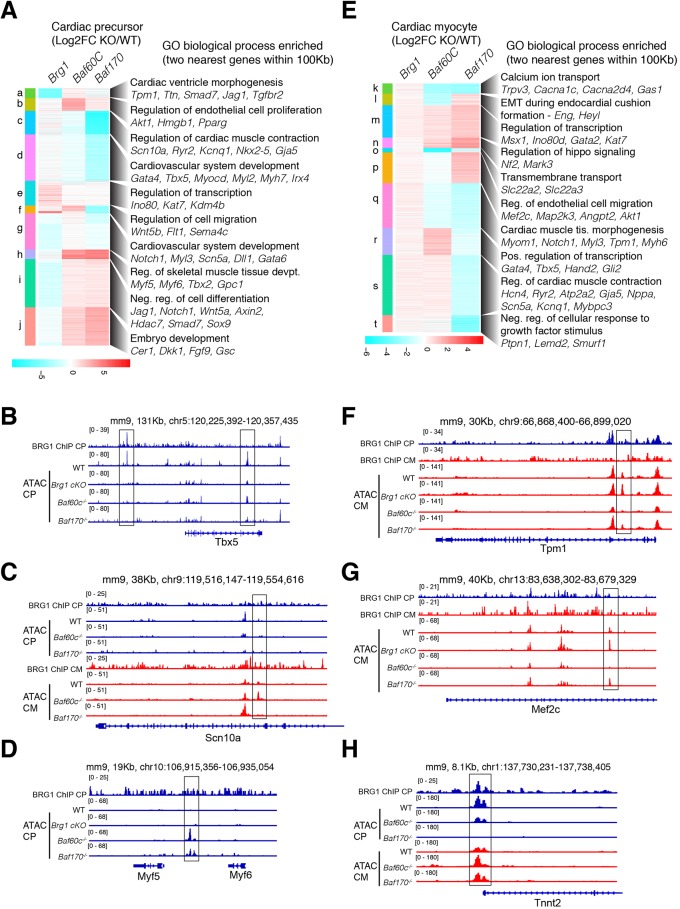
**BRG1, BAF60c and BAF170 regulate temporal chromatin accessibility.** (A,E) Heat maps of significantly affected ATAC-seq peaks (FDR <0.05, twofold change) in BRG1 conditional, BAF60c KO and BAF170 KO over their respective wild-type counterparts in CPs (A) or CMs (E). Enriched biological processes of the two nearest genes (within 100 kb) to the ATACseq peaks were analyzed using GREAT (McLean et al., 2010) and are shown to the right with representative genes. (C,H) Browser tracks showing delayed opening of *Tnnt2* promoter chromatin (H) and loss of *Scn10a* enhancer in absence of BAF170 (C). (B,D,F,G) Chromatin opening and closing near *Tbx5* (B) and *Myf5* (D) in the absence of BAF60c and BAF170 in CPs, and chromatin closing in *Tpm1* (F) and *Mef2c* (G) in the absence of BAF60c in CMs are shown.

To understand the function of BRG1, BAF60c and BAF170 in gene expression regulation, we used ATACseq (Corces et al., 2017) to examine chromatin accessibility genome wide in CPs and CMs. We compared differential chromatin accessibility profiles in *Brg1* conditional KOs, and *Baf60c* and *Baf170* KOs in CP and CM cells, and analyzed the two nearest (within 100 kb) genes to an ATACseq site, for enrichment of biological processes (Fig. 4). In CPs, *Brg1* maintains chromatin accessibility near genes involved in cardiovascular development, cardiac tissue morphogenesis and regulation of cell differentiation (Fig. 4A, clusters *a*, *g* and *i*), and prevents chromatin accessibility near genes involved in transcriptional regulation and chromatin organization (Fig. 4A, cluster *e*). In the absence of BAF60c, accessibility was increased at genes that are involved in non-cardiac cell differentiation and regulation of cell migration (Fig. 4A, cluster *f*). In contrast, in the absence of either BAF60c or BAF170, accessibility was reduced near genes involved in cardiovascular development (*Gata4*, *Tbx5*, *Myocd* and *Myh7*), calcium handing (*Ryr2*) and muscle contraction (*Scn5a*, *Scn10a* and *Kcnq1*) (Fig. 4A, clusters c and d; Fig. 4B,C). Loss of BAF60c and BAF170 increased chromatin accessibility near genes involved with embryonic limb and skeletal muscle development (*Myf5*, *Myf6* and *Tbx2*) and early embryo development (*Cer1*, *Dkk1*, *Fgf9* and *Gsc*) (Fig. 4A, cluster *h-j* and Fig. 4D).

In CMs, loss of BRG1 did not change chromatin structure, consistent with gene expression (Figs 4E and 1C), whereas loss of BAF60c and BAF170 increased chromatin accessibility near genes involved in chromatin and transcription regulation (*Ino80d* and *Kat7*), and hematopoietic differentiation (*Gata2*). BAF60c alone is implicated in promoting chromatin accessibility near CP genes (*Gata4*, *Tbx5* and *Hand2*), whereas BAF60c and BAF170 are important in chromatin accessibility near genes involved in cardiac function (*Myom1*, *Tpm1*, *Myh6* and *Myl3*) (Fig. 4F) and calcium ion transport (*Cacna1c* and *Cacna2d4*). Uniquely, they also regulate chromatin near signaling and cell migration genes (Fig. 4G). Furthermore, in the absence of BAF170, we observed delayed accessibility of the *Tnnt2* promoter (Fig. 4H), consistent with delayed onset of beating (Fig. 3C), and impaired accessibility at an enhancer in the *Scn10a* gene (Fig. 4C). This *Scn10a* region is a known TBX5-dependent enhancer of *Scn5a*, and was identified as containing a GWAS SNP associated with altered electrophysiology in humans (van den Boogaard et al., 2014, 2012). These data provide evidence of the unique and shared functions that individual BAF subunits exert in order to regulate chromatin structure.

### Both BAF170 and BAF60c regulate BAF complex composition

**Fig. 5.**
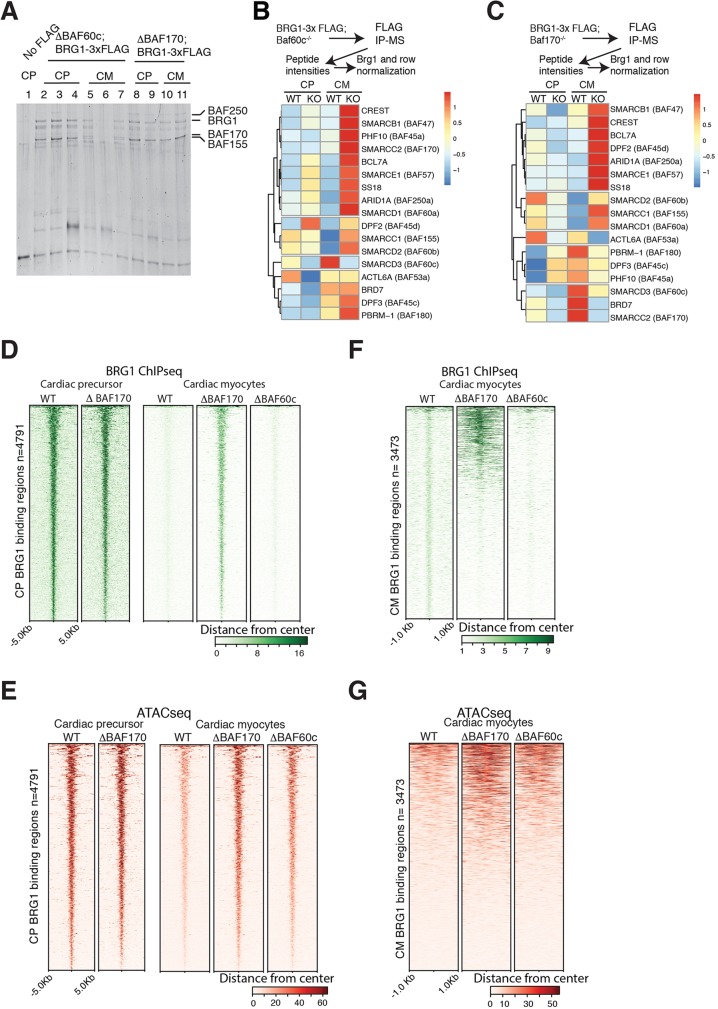
**Cardiac-enriched BAF subunits regulate BRG1 complex relative abundance and genome binding.** (A) An immunoprecipitated and FLAG-eluted BRG1-containing complex isolated from untagged cells or from cells lacking BAF60c or BAF170 in CPs and CMs analyzed using 10% SDS-PAGE. (B,C) Peptide intensities of FLAG immunopurification-mass spectrometry in the absence of BAF60c (B) or BAF170 (C) in CPs and CMs after normalization to mock control and to BRG1 levels, and across stages of differentiation. (D) ChIP-Seq showing BRG1 binding over 4791 peaks in wild-type and BAF170 KO CPs (left panel). BRG1 binding over these same 4791 CP sites in wild-type, BAF170 KO or BAF60c KO CMs (right panel). (E) ATACseq signal over 4791 BRG1-binding sites in CPs and CMs for the indicated genotypes. (F) BRG1 ChIP over 3473 peaks in wild-type, BAF170 KO and BAF60c KO CMs. (G) ATACseq signal over 3473 CM BRG1-binding sites over the indicated genotypes in CMs.

BAF60c and BAF170 have crucial roles in regulation of chromatin structure and transcription. It is not clear whether mammalian BAF complex composition is reliant on the presence of specific subunits. Importantly, BAF60 and BAF170 family subunits are part of the initiating steps in BAF complex assembly (Mashtalir et al., 2018). To understand the nature of BAF complexes formed in the absence of these cardiac-enriched subunits, we immunopurified BRG1-3×FLAG complexes from ESC lines lacking BAF60c or BAF170 at CP and CM stages. SDS-PAGE revealed increased abundance of BRG1-containing complexes in BAF170 KO CMs (Fig. 5A). During the CP to CM transition, BRG1 complex abundance is normally reduced in wild-type cells (Fig. 2A, compare lane 5 with lane 6) and in BAF60c KO cells (Fig. 5A, compare lanes 2-4 with lanes 5 and 7). In BAF170 KO the abundance of BAF complexes remained unchanged (Fig. 5A, compare lanes 8 and 9 with lanes 10 and 11), indicating a crucial role for BAF170 in BRG1 complex abundance.

MS analyses of these complexes revealed significant differences in subunit composition in the absence of BAF60c or BAF170 (Fig. 5B,C). In CPs, BRG1 complexes lacking BAF60c had reduced association of BAF53a, BRD7, BAF45a and BAF45c, and were enriched for BAF45d, BAF47, SS18, BAF155 and BAF60a (Fig. 5B and Fig. S4A). In CMs, we observed increased association of many subunits with BRG1 in the absence of BAF60c (Fig. 5B, Fig. S4A and S4B). Similarly, BAF170 loss led to BRG1-containing complexes with reduced association of BCL7a, BAF45d, BAF60c, BAF47 and BAF53a (ACTL6A), and increased association of BAF45c, SS18L1 (CREST) and BAF180 (PRBM1) with BRG1 in CPs. In CMs, loss of BAF170 reduced association with BAF60c and increased association with BAF155 and BAF60b (Fig. 5C, Fig. S4A and S4B). The altered complexes formed are not due to changes in the transcriptional level of BAF subunits (Fig. S4C).

These results suggest that a fine balance exists in the composition of the BAF complex and perturbation of one subunit extends to the association of other subunits in the complex. It further indicates that different subunits or isoforms substitute for the absence of one or more subunits. For example, both BAF60a and BAF60b substitute for the lack of BAF60c, and depletion of BAF45c is balanced by enrichment of BAF45a (Fig. S4B). These results emphasize that subunit switching and substitution in BAF complex composition could be an important mechanism in cardiac lineage specification.

### BAF170 facilitates temporal BRG1 dissociation from the genome

We explored the possibility that BAF60c or BAF170 help direct the genomic localization of BRG1. In CPs, BRG1 binds to a set of 4791 sites (Fig. S1B) and these are largely unaffected in absence of BAF170 (Fig. 5D). BRG1 binding at these site is severely reduced in CMs, perhaps owing to the reduced abundance of BRG1-containing complexes (Fig. 2A, compare lane 5 with 6; Fig. 2E, BRG1 western, compare CP with CM). However, in the absence of BAF170, BRG1 binding at a large subset of these sites is retained in CMs (Fig. 5D), consistent with the increased relative abundance of BRG1-containing complexes. These chromatin regions are accessible in CPs and are normally subsequently inaccessible in CMs, but remain accessible in absence of BAF170, consistent with retained BRG1 complexes remodeling these regions (Fig. 5E). In CMs, BRG1 weakly bound to 3473 sites, and lack of BAF170 increased BRG1 binding to some of these (Fig. 5F), whereas the absence of BAF60c reduced the binding of BRG1. These binding dynamics correlated with chromatin accessibility (Fig. 5G). This suggests that recruitment of BRG1-inclusive complexes is regulated by cardiac-specific subunits, and that concomitant dynamic expulsion of the complex is also highly regulated by similar processes.

## DISCUSSION

BRG1-containing complexes respond to and modulate lineage decisions and differentiation in cardiac development by incorporating specific subunits at discrete stages. These specialized BAF complexes regulate distinct gene expression programs to drive cardiogenesis while simultaneously repressing alternate non-cardiac developmental programs. Our results suggest that BAF complexes use multiple interdependent mechanisms, including switching subunits or isoforms, regulating relative subunit abundance, altering BRG1 recruitment and expulsion strategies, to modulate dynamics of cardiac differentiation. This unanticipated complexity of chromatin complex regulation emerges as a crucial determinant of differentiation.

Subunit composition switching of chromatin remodeling complexes have been reported during neural development (Lessard et al., 2007; Nitarska et al., 2016; Yoo et al., 2009) and skeletal myogenesis (Goljanek-Whysall et al., 2014; Saccone et al., 2014). For example, during myogenic differentiation, BAF60c expression is facilitated over the alternative isoforms BAF60a and BAF60b, which are post-transcriptionally repressed by myogenic microRNAs. However, the systematic immunopurification-MS-based discovery of BAF/PBAF complex subunits during the time course of a well-regulated differentiation process has, to date, not been achieved. Importantly, we show that BRG1-associated subunits at different stages of cardiac differentiation form specific complexes to activate or repress specific transcriptional programs. In addition, we determine that the presence of specific BAF subunits greatly dictates the overall composition, and therefore function, of the complex.

The continued roles played by BAF60c and BAF170 are intriguing considering the reduced role of BRG1 in later stages of cardiac differentiation. The incorporation of these subunits within a BRM-containing complex could explain this, although the apparent absence of a developmental or postnatal cardiac phenotype in BRM-null mice might indicate minimal importance of these complexes (Reyes et al., 1998). It has been suggested that BAF60c may function independently of the BAF complex (Forcales et al., 2012; Wang et al., 2013), although there is no evidence for this in cardiac cells. It is also possible that the altered BAF complex assembly in the absence of BAF60c or BAF170 creates a set of complexes with anomalous function, thus leading to aberrant gene expression.

BAF60c and BAF170 are essential for BRG1 complex composition. Altered association or dissociation of several subunits in the absence of BAF60c or BAF170 indicates the presence of sub-modules within the BAF complex mediated by these subunits. Our observations are consistent with reports in yeast SWI/SNF complexes where the absence of specific subunits forms sub-modules or altered SWI/SNF complex composition (Dutta et al., 2017; Sen et al., 2017; Yang et al., 2007). Thus, phenotypic observations in absence of a particular subunit may be the result of disruption of a module containing many subunits. Conflicting evidence regarding this mechanism has been shown for other BAF complex subunits in mammalian cells (Nakayama et al., 2017; Wang et al., 2017). We interpret our results as indicating that a stable complex with broadly altered composition exists in the absence of BAF60c or BAF170.

BRG1 binding to genomic sites in CPs and putative BRG1 targets both have crucial roles in the promotion of cardiac gene expression programs, while repressing non-cardiac fate. BRG1 binding is greatly reduced in CMs, indicating diminished BRG1 function. Our finding that BAF170 promotes BRG1 dissociation from the genome indicates a potential mechanism of BAF170-mediated gene regulation during differentiation. How BAF170 regulates genomic expulsion of BRG1 is currently not understood, but association of BAF170 with BRM-containing complexes in CM might evict BRG1 from BAF complexes as both BRG1 and BRM are mutually exclusive. Furthermore, a concomitant increase in BAF170 and decrease in BAF155 association with BRG1-containing complexes during cardiac differentiation might make BRG1 prone to post-transcriptional modification and proteasome-mediated degradation, as BAF155 is known to stabilize BRG1-containing complexes (Sohn et al., 2007). Consistently, we observe increase in BRG1 complex abundance in the absence of BAF170 in CMs.

In conclusion, our study identifies morphing combinations of specific BAF subunits that form and change subunit compositions during cardiac differentiation, and drive stage-specific cardiac gene expression programs. These results are consistent with the known *in vivo* roles of BAF complex subunits (Hang et al., 2010; Sun et al., 2018; Takeuchi et al., 2011). Genetic dissection of individual subunit contribution clearly unveils a profound and specific function of individual BAF complex subunits. How BAF chromatin remodelers with different subunit composition provide specificity to gene expression programs will be the focus of future studies.

## MATERIALS AND METHODS

### Cardiomyocyte differentiation

Mouse embryonic stem cells (ESCs) were cultured in feeder-free condition sin serum and leukemia inhibitory factor-containing medium. CMs were differentiation as described previously (Kattman et al., 2011; Wamstad et al., 2012). *Brg1* was deleted in presence of 200 nM 4-hydroxytamoxifen for 48 h with control cells treated similarly with tetrahydrofuran (Ho et al., 2009a).

### Knockout cell line generation

BAF60c was inactivated by targeting exon 2 of *Baf60c* with two pairs of TALENs following the methods of Sanjana et al. (2012), except a CAGGS promoter replaced the CMV promoter (Giorgetti et al., 2014). The targets of TALEN are as follows: GCCCCCTAAGCCCTCTCCAGAGAACATCCAAGCTA GAATGACTTGGTCGCTGCTAC and CCCGCCCCTCTCCAAGACCCTGGGTTGGTA ACCCTGCGCTGAGCGATGAGTGGGAG.

BAF170 was targeted using CRISPR/Cas9 with sgRNA targeting exon 2 of *Baf170* following the protocol described by Cong et al. (2013). sgRNA were cloned to a *Bbs*I-digested pX330 vector by annealing the following primers: 5′ caccg CGCACCGCTTACTAAACTGC 3′ and 5′ aaac GCAGTTTAGTAAGCGGTGCG c 3′ (lowercase indicates the *Bbs*I digestion site). Targeting vector was constructed by cloning 458 and 459 bp of Baf170 DNA upstream and downstream of midpoint of sgRNA target site into *Kpn*I-*Xho*I and *Bam*H1-*Not*I sites of pFPF (a derivative of Addgene plasmid #22678 in which neomycin is replaced with puromycin cassette). 2.5 μg of each TALEN Baf60c plasmid or the sgRNA plasmid, plus 20 μg of BAF170 targeting constructs were used for transfection. Single clones were selected, grown, PCR genotyped and DNA sequenced.

### Nuclear extract preparation, western blot and anti-FLAG immunoaffinity purification

Nuclear extracts were prepared using protocols described previously (Abmayr et al., 2006). Western blotting was performed using standard techniques with PVDF membranes. Primary antibodies used were anti-BRG1 (Abcam, ab110641, 1:1000), anti-FLAG (Sigma, F1804, 1:1000), anti-BAF155 (Bethyl, A301-021A, 1:1000), anti-BAF170 (Bethyl, 1:1000, A301-39A), anti-BAF60c (Cell Signaling Technology, 62265, 1:1000) and anti-WDR5 (Bethyl, A302-430A, 1:2000 dilution). Secondary antibodies used were donkey anti-rabbit IRDye 800cw (Licor, 926-32213, 1:10,000), donkey anti-mouse IRDye 800cw (Licor, 925-32212, 1:10,000) and donkey anti-goat IRDye 680cw (Licor, 925-68074-1:10,000).

For immunoaffinity purification of BAF complexes, we leached out nuclear protein by using high-salt and glycerol, and did not digest DNA or RNA. Cell pellets (10^8^ cells) were incubated in a hypotonic buffer (10 mM TrisCl, 10 mM NaCl) and centrifuged at 500 ***g*** to separate the cytoplasmic fraction from nuclei. Nuclear proteins were leached out in nuclear extract buffer containing 0.3 M NaCl, 0.02% NP40 and 20% glycerol by rotating for 1 h at 4°C in absence of DNase or RNase. Soluble nuclear extracts were separated by centrifuging at 100,000 ***g*** for 1 h. The nuclear extract was incubated with 50 μl of anti-FLAG M2 agarose gel (Sigma, A2220) overnight, washed 10 times with the same nuclear buffer containing 0.3 M NaCl, 0.02% NP40 and 20% glycerol, and batch eluted with 0.1 mg/ml FLAG peptides (ELIM Biopharmaceutical). Protein complexes were resolved using 10% SDS PAGE.

### Mass spectrometry

Protein complexes were digested with trypsin for LC-MS/MS analysis. Samples were denatured and reduced in 2 M urea, 10 mM NH_4_HCO_3_, 2 mM DTT for 30 min at 60°C, then alkylated with 2 mM iodoacetamide for 45 min at room temperature. Trypsin (Promega) was added at a 1:100 enzyme:substrate ratio and digested overnight at 37°C. Following digestion, samples were concentrated using C18 ZipTips (Millipore) according to the manufacturer's specifications. Desalted samples were evaporated to dryness and resuspended in 0.1% formic acid for mass spectrometry analysis.

Digested peptide mixtures were analyzed by LC-MS/MS on a Thermo Fisher Scientific LTQ Orbitrap Elite mass spectrometry system equipped with a Proxeon Easy nLC 1000 ultra high-pressure liquid chromatography and autosampler system. Samples were injected onto a C18 column (25 cm×75 μm I.D. packed with ReproSil Pur C18 AQ 1.9 µm particles) in 0.1% formic acid and then separated with a 1 h gradient from 5% to 30% ACN in 0.1% formic acid at a flow rate of 300 nl/min. The mass spectrometer collected data in a data-dependent fashion, collecting one full scan in the Orbitrap at 120,000 resolution followed by 20 collision-induced dissociation MS/MS scans in the dual linear ion trap for the 20 most intense peaks from the full scan. Dynamic exclusion was enabled for 30 s with a repeat count of 1. Charge state screening was employed to reject analysis of singly charged species or species for which a charge could not be assigned.

Raw mass spectrometry data were analyzed using the MaxQuant software package (version 1.3.0.5) (Cox and Mann, 2008). Data were matched to the SwissProt mouse protein sequences (downloaded from UniProt on 19 July 2016). MaxQuant was configured to generate and search against a reverse sequence database for false discovery rate (FDR) calculations. Variable modifications were allowed for methionine oxidation and protein N-terminus acetylation. A fixed modification was indicated for cysteine carbamidomethylation. Full trypsin specificity was required. The first search was performed with a mass accuracy of ±20 ppm and the main search was performed with a mass accuracy of ±6 ppm. A maximum of five modifications were allowed per peptide. A maximum of two missed cleavages were allowed. The maximum charge allowed was 7+. Individual peptide mass tolerances were allowed. For MS/MS matching, a mass tolerance of 0.5 Da was allowed and the top six peaks per 100 Da were analyzed. MS/MS matching was allowed for higher charge states, water and ammonia loss events. The data were filtered to obtain a peptide, protein and site-level FDR of 0.01. The minimum peptide length was seven amino acids. Results were matched between runs with a time window of 2 min for technical duplicates. All precursor (MS1) intensities of valid peptide matches were quantified by the Maxquant LFQ algorithm using the match between runs option to minimize missing values.

For statistical analysis, the quantitative change of peptides that were uniquely assigned to protein isoforms across all immunopurifications were compared with the MSstats R package (v. 2.3.4) (Choi et al., 2014). Briefly, all peak intensities were Log_2_-transformed and their distributions were median centered across all runs using the scale option. All remaining missing intensity values were imputed by setting their value to minimal intensity value per run, as an estimate for the MS Limit Of Quantitation. The normalized dataset was then analyzed by fitting a mixed effects model per protein using the model without interaction terms, unequal feature variance, and restricted scope of technical and biological replication. The average change (Log2-Fold-Change) of the model-based abundance estimate was computed by comparing replicates of each differentiation stage against the ESC undifferentiated pool. Proteins with a greater than fourfold change (Log2 Fold Change >2) and test *P*-value<0.05 were determined as significantly altered during differentiation. The mass spectrometry proteomics data have been deposited to the ProteomeXchange Consortium via the PRIDE (Perez-Riverol et al., 2018) partner repository with the dataset identifier PXD012721.

### Nucleosome reconstitution, repositioning and ATPase assay

Recombinant *Xenopus laevis* histone octamers were reconstituted on a 601-nucleosome positioning DNA (Lowary and Widom, 1998) as described previously (Hota et al., 2013). Nucleosome repositioning and ATP hydrolysis assays were performed as described previously (Hota et al., 2013).

### RNA-sequencing

Total RNA was isolated from biologically triplicate samples using miRNeasy micro kit with on-column DNase I digestion (Qiagen). RNA-seq libraries were prepared using the Ovation RNA-seq system v2 kit (NuGEN). Libraries from the SPIA amplified cDNA were made using the Ultralow DR library kit (NuGEN). RNA-seq libraries were analyzed using Bioanalyzer, quantified using KAPA QPCR and paired-end 100 bp reads were sequenced using a HiSeq 2500 instrument (Illumina). RNA reads were aligned with TopHat2 (Kim et al., 2013), counts per gene calculated using feature Counts (Liao et al., 2014) and edgeR (Robinson et al., 2010) was used for the analysis of differential expression. K-means clustering and pheatmap functions in R were used to cluster and generate heatmaps. GO enrichment analysis were performed using GO_Elite (Zambon et al., 2012).

### ChIP-Seq

Chromatin immunoprecipitations were performed according to O'Geen et al. (2011). Briefly, cells were double crosslinked with 2 mM disuccinimidyl glutarate (DSG) and 1% formaldehyde, and quenched with 0.125 M glycine. Frozen pellets (5×10^7^) were thawed, washed, dounced and digested with MNase. Chromatin was sonicated at output 4 for 30 s twice with a 1 min pause between cycles then centrifuged at 10,000 ***g*** for 10 min at 4°C and stored at −80°C. Chromatin (40 μg) was diluted to fivefold, pre-cleared for 2 h followed by immunoprecipitation with anti-Brg1 antibody (Abcam, 110641) for 12-16 h. 5% of samples were used as input DNA. Antibody-bound BRG1-DNA complexes were immunoprecipitated using 25 μl of M-280 goat anti-rabbit IgG dyna beads for 2 h, washed a total of ten times with buffers [twice with IP wash buffer 1 containing 50 mM Tris.Cl (pH 7.4), 150 mM NaCl, 1% NP-40, 0.25% sodium deoxycholate and 1 mM EDTA), five times with IP wash buffer 2 containing 100 mM Tris.Cl (pH 9.0), 500 mM LiCl, 1% NP-40 and 1% sodium deoxcholate, and then twice with IP wash buffer 2 along with 150 mM NaCl] of increasing stringency and eluted with 200 μl of elution buffer [10 mM TrisCl (pH 7.5), 1 mM EDTA and 1%SDS). Samples were reverse crosslinked, digested with proteinase K and RNAse A, and purified using AMPure XP beads (Beckman Coulter). To prepare libraries for sequencing, DNA was end repaired, A-tailed, adapter ligated (Illumina TrueSeq) and PCR amplified. PCR-amplified libraries were size selected and ampure purified. The concentration and size of eluted libraries was measured (Qubit and Bioanalyzer) before sequencing using a NEBNextSeq sequencer.

Reads (single end 75 bp) were trimmed using fastq-mcf and aligned to mouse genome mm9 assembly using Bowtie (Langmead et al., 2009). Minimum mapping quality score was set to 30. Statistically enriched bins with a *P*-value threshold set to 1×10^6^ were determined using input DNA as the background model (Thomas et al., 2017). Galaxy (usegalaxy.org/) was used to pool multiple replicates to generate browser tracks and tornado plots. GREAT (McLean et al., 2010) was used to generate gene lists near BRG1 peaks. The bioconductor package ‘annotatr’ version 3.8 (Cavalcante and Sartor, 2017) was used to classify BRG1-binding sites within promoters (<1 kb from the transcription start site), introns, intergenic regions and exons. The HOMER (Heinz et al., 2010) motif enrichment package was used to enrich DNA motifs in BRG1-binding sites. HOMER calculates the q-value of known motifs to statistically confirm to Benjamini-Hochberg multiple hypothesis testing corrections.

### ATAC sequencing

Assay for transposase-accessible chromatin using sequencing (ATAC-seq) was performed according to Corces et al. (2017) in two to five biological replicates. Briefly, 50,000 cells (>95% viability) were lysed, washed and tagmented for 45 mins and 3 h for CP and CM cells, respectively. DNA was purified and amplified using universal Ad1 and barcoded reverse primers (Corces et al., 2017). Libraries were purified, quantified and analyzed on a bioanalyzer and sequenced on a NEB NextSeq 550 sequencer using Illumina NextSeq 500/550 High Output v2 kit (150 cycles). Sequencing image files were de-multiplexed and fastq generated. Reads (paired end 75 bp) were trimmed and aligned to mouse genome mm9 assembly using Bowtie (Langmead et al., 2009) with a minimum mapping quality score of 30. Statistically enriched bins with a *P*-value threshold set to 1×10^6^ were determined (Thomas et al., 2017). UCSC genome browser and IGV were used to view the browser tracks. Galaxy (usegalaxy.org) was used to pool multiple replicates to generate browser tracks and tornado plots. GREAT was used to generate gene lists near ATACseq sites.

## Supplementary Material

Supplementary information

## References

[DEV174086C1] Abmayr S. M., Yao T., Parmely T. and Workman J. L. (2006). Preparation of nuclear and cytoplasmic extracts from mammalian cells. *Curr. Protoc. Mol. Biol.* Chapter 12, 12.1-12.10. 10.1002/0471142727.mb1201s75 18265374

[DEV174086C2] Alexander J. M., Hota S. K., He D., Thomas S., Ho L., Pennacchio L. A. and Bruneau B. G. (2015). Brg1 modulates enhancer activation in mesoderm lineage commitment. *Development* 142, 1418-1430. 10.1242/dev.109496 25813539 PMC4392595

[DEV174086C3] Attanasio C., Nord A. S., Zhu Y., Blow M. J., Biddie S. C., Mendenhall E. M., Dixon J., Wright C., Hosseini R., Akiyama J. A. et al. (2014). Tissue-specific SMARCA4 binding at active and repressed regulatory elements during embryogenesis. *Genome Res.* 24, 920-929. 10.1101/gr.168930.113 24752179 PMC4032856

[DEV174086C4] Bartholomew B. (2014). Regulating the chromatin landscape: structural and mechanistic perspectives. *Annu. Rev. Biochem.* 83, 671-696. 10.1146/annurev-biochem-051810-093157 24606138 PMC4332854

[DEV174086C5] Cavalcante R. G. and Sartor M. A. (2017). annotatr: genomic regions in context. *Bioinformatics* 33, 2381-2383. 10.1093/bioinformatics/btx183 28369316 PMC5860117

[DEV174086C6] Chang C.-P. and Bruneau B. G. (2012). Epigenetics and cardiovascular development. *Annu. Rev. Physiol.* 74, 41-68. 10.1146/annurev-physiol-020911-153242 22035349

[DEV174086C7] Choi M., Chang C.-Y., Clough T., Broudy D., Killeen T., MacLean B. and Vitek O. (2014). MSstats: an R package for statistical analysis of quantitative mass spectrometry-based proteomic experiments. *Bioinformatics* 30, 2524-2526. 10.1093/bioinformatics/btu305 24794931

[DEV174086C8] Clapier C. R. and Cairns B. R. (2009). The biology of chromatin remodeling complexes. *Annu. Rev. Biochem.* 78, 273-304. 10.1146/annurev.biochem.77.062706.153223 19355820

[DEV174086C9] Cong L., Ran F. A., Cox D., Lin S., Barretto R., Habib N., Hsu P. D., Wu X., Jiang W., Marraffini L. A. et al. (2013). Multiplex genome engineering using CRISPR/Cas systems. *Science* 339, 819-823. 10.1126/science.1231143 23287718 PMC3795411

[DEV174086C10] Corces M. R., Trevino A. E., Hamilton E. G., Greenside P. G., Sinnott-Armstrong N. A., Vesuna S., Satpathy A. T., Rubin A. J., Montine K. S., Wu B. et al. (2017). An improved ATAC-seq protocol reduces background and enables interrogation of frozen tissues. *Nat. Meth.* 14, 959-962. 10.1038/nmeth.4396 PMC5623106 28846090

[DEV174086C11] Cox J. and Mann M. (2008). MaxQuant enables high peptide identification rates, individualized p.p.b.-range mass accuracies and proteome-wide protein quantification. *Nat. Biotechnol.* 26, 1367-1372. 10.1038/nbt.1511 19029910

[DEV174086C12] Davidson E. H. (2010). Emerging properties of animal gene regulatory networks. *Nature* 468, 911-920. 10.1038/nature09645 21164479 PMC3967874

[DEV174086C13] Devine W. P., Wythe J. D., George M., Koshiba-Takeuchi K. and Bruneau B. G. (2014). Early patterning and specification of cardiac progenitors in gastrulating mesoderm. *Elife* 3, 508 10.7554/eLife.03848 PMC4356145 25296024

[DEV174086C14] Dutta A., Sardiu M., Gogol M., Gilmore J., Zhang D., Florens L., Abmayr S. M., Washburn M. P. and Workman J. L. (2017). Composition and function of mutant Swi/Snf complexes. *Cell Rep.* 18, 2124-2134. 10.1016/j.celrep.2017.01.058 28249159 PMC5837817

[DEV174086C15] Evans S. M., Yelon D., Conlon F. L. and Kirby M. L. (2010). Myocardial lineage development. *Circ. Res.* 107, 1428-1444. 10.1161/CIRCRESAHA.110.227405 21148449 PMC3073310

[DEV174086C16] Forcales S. V., Albini S., Giordani L., Malecova B., Cignolo L., Chernov A., Coutinho P., Saccone V., Consalvi S., Williams R. et al. (2012). Signal-dependent incorporation of MyoD-BAF60c into Brg1-based SWI/SNF chromatin-remodelling complex. *EMBO J.* 31, 301-316. 10.1038/emboj.2011.391 22068056 PMC3261556

[DEV174086C17] Giorgetti L., Galupa R., Nora E. P., Piolot T., Lam F., Dekker J., Tiana G. and Heard E. (2014). Predictive polymer modeling reveals coupled fluctuations in chromosome conformation and transcription. *Cell* 157, 950-963. 10.1016/j.cell.2014.03.025 24813616 PMC4427251

[DEV174086C18] Goljanek-Whysall K., Mok G. F., Fahad Alrefaei A., Kennerley N., Wheeler G. N. and Münsterberg A. (2014). myomiR-dependent switching of BAF60 variant incorporation into Brg1 chromatin remodeling complexes during embryo myogenesis. *Development* 141, 3378-3387. 10.1242/dev.108787 25078649 PMC4199139

[DEV174086C19] Hang C. T., Yang J., Han P., Cheng H.-L., Shang C., Ashley E., Zhou B. and Chang C.-P. (2010). Chromatin regulation by Brg1 underlies heart muscle development and disease. *Nature* 466, 62-67. 10.1038/nature09130 20596014 PMC2898892

[DEV174086C20] Heinz S., Benner C., Spann N., Bertolino E., Lin Y. C., Laslo P., Cheng J. X., Murre C., Singh H. and Glass C. K. (2010). Simple combinations of lineage-determining transcription factors prime cis-regulatory elements required for macrophage and B cell identities. *Mol. Cell* 38, 576-589. 10.1016/j.molcel.2010.05.004 20513432 PMC2898526

[DEV174086C21] Ho L. and Crabtree G. R. (2010). Chromatin remodelling during development. *Nature* 463, 474-484. 10.1038/nature08911 20110991 PMC3060774

[DEV174086C22] Ho L., Jothi R., Ronan J. L., Cui K., Zhao K. and Crabtree G. R. (2009a). An embryonic stem cell chromatin remodeling complex, esBAF, is an essential component of the core pluripotency transcriptional network. *Proc. Natl. Acad. Sci. USA* 106, 5187-5191. 10.1073/pnas.0812888106 19279218 PMC2654397

[DEV174086C23] Ho L., Ronan J. L., Wu J., Staahl B. T., Chen L., Kuo A., Lessard J., Nesvizhskii A. I., Ranish J. and Crabtree G. R. (2009b). An embryonic stem cell chromatin remodeling complex, esBAF, is essential for embryonic stem cell self-renewal and pluripotency. *Proc. Natl. Acad. Sci. USA* 106, 5181-5186. 10.1073/pnas.0812889106 19279220 PMC2654396

[DEV174086C24] Ho L., Miller E. L., Ronan J. L., Ho W. Q., Jothi R. and Crabtree G. R. (2011). esBAF facilitates pluripotency by conditioning the genome for LIF/STAT3 signalling and by regulating polycomb function. *Nat. Cell Biol.* 13, 903-913. 10.1038/ncb2285 21785422 PMC3155811

[DEV174086C25] Hota S. K. and Bruneau B. G. (2016). ATP-dependent chromatin remodeling during mammalian development. *Development* 143, 2882-2897. 10.1242/dev.128892 27531948 PMC5004879

[DEV174086C26] Hota S. K., Bhardwaj S. K., Deindl S., Lin Y.-C., Zhuang X. and Bartholomew B. (2013). Nucleosome mobilization by ISW2 requires the concerted action of the ATPase and SLIDE domains. *Nat. Struct. Mol. Biol.* 20, 222-229. 10.1038/nsmb.2486 23334290 PMC3565048

[DEV174086C27] Jin S. C., Homsy J., Zaidi S., Lu Q., Morton S., DePalma S. R., Zeng X., Qi H., Chang W., Sierant M. C. et al. (2017). Contribution of rare inherited and de novo variants in 2,871 congenital heart disease probands. *Nat. Genet.* 49, 1593-1601. 10.1038/ng.3970 28991257 PMC5675000

[DEV174086C28] Kadoch C. and Crabtree G. R. (2013). Reversible disruption of mSWI/SNF (BAF) complexes by the SS18-SSX oncogenic fusion in synovial sarcoma. *Cell* 153, 71-85. 10.1016/j.cell.2013.02.036 23540691 PMC3655887

[DEV174086C29] Kattman S. J., Witty A. D., Gagliardi M., Dubois N. C., Niapour M., Hotta A., Ellis J. and Keller G. (2011). Stage-specific optimization of activin/nodal and BMP signaling promotes cardiac differentiation of mouse and human pluripotent stem cell lines. *Cell Stem Cell* 8, 228-240. 10.1016/j.stem.2010.12.008 21295278

[DEV174086C30] Kim D., Pertea G., Trapnell C., Pimentel H., Kelley R. and Salzberg S. L. (2013). TopHat2: accurate alignment of transcriptomes in the presence of insertions, deletions and gene fusions. *Genome Biol.* 14, R36 10.1186/gb-2013-14-4-r36 23618408 PMC4053844

[DEV174086C31] Langmead B., Trapnell C., Pop M. and Salzberg S. L. (2009). Ultrafast and memory-efficient alignment of short DNA sequences to the human genome. *Genome Biol.* 10, R25 10.1186/gb-2009-10-3-r25 19261174 PMC2690996

[DEV174086C32] Lessard J., Wu J. I., Ranish J. A., Wan M., Winslow M. M., Staahl B. T., Wu H., Aebersold R., Graef I. A. and Crabtree G. R. (2007). An essential switch in subunit composition of a chromatin remodeling complex during neural development. *Neuron* 55, 201-215. 10.1016/j.neuron.2007.06.019 17640523 PMC2674110

[DEV174086C33] Liao Y., Smyth G. K. and Shi W. (2014). featureCounts: an efficient general purpose program for assigning sequence reads to genomic features. *Bioinformatics* 30, 923-930. 10.1093/bioinformatics/btt656 24227677

[DEV174086C34] Lickert H., Takeuchi J. K., von Both I., Walls J. R., McAuliffe F., Adamson S. L., Henkelman R. M., Wrana J. L., Rossant J. and Bruneau B. G. (2004). Baf60c is essential for function of BAF chromatin remodelling complexes in heart development. *Nature* 432, 107-112. 10.1038/nature03071 15525990

[DEV174086C35] Loh K. M., Chen A., Koh P. W., Deng T. Z., Sinha R., Tsai J. M., Barkal A. A., Shen K. Y., Jain R., Morganti R. M. et al. (2016). Mapping the pairwise choices leading from pluripotency to human bone, heart, and other mesoderm cell types. *Cell* 166, 451-467. 10.1016/j.cell.2016.06.011 27419872 PMC5474394

[DEV174086C36] Lowary P. T. and Widom J. (1998). New DNA sequence rules for high affinity binding to histone octamer and sequence-directed nucleosome positioning. *J. Mol. Biol.* 276, 19-42. 10.1006/jmbi.1997.1494 9514715

[DEV174086C37] Luna-Zurita L., Stirnimann C. U., Glatt S., Kaynak B. L., Thomas S., Baudin F., Samee M. A. H., He D., Small E. M., Mileikovsky M. et al. (2016). Complex interdependence regulates heterotypic transcription factor distribution and coordinates cardiogenesis. *Cell* 164, 999-1014. 10.1016/j.cell.2016.01.004 26875865 PMC4769693

[DEV174086C38] Mashtalir N., D'Avino A. R., Michel B. C., Luo J., Pan J., Otto J. E., Zullow H. J., McKenzie Z. M., Kubiak R. L., Pierre R. S. et al. (2018). Modular organization and assembly of SWI/SNF family chromatin remodeling complexes. *Cell* 175, 1272-1288.e20. 10.1016/j.cell.2018.09.032 30343899 PMC6791824

[DEV174086C39] McLean C. Y., Bristor D., Hiller M., Clarke S. L., Schaar B. T., Lowe C. B., Wenger A. M. and Bejerano G. (2010). GREAT improves functional interpretation of cis-regulatory regions. *Nat. Biotechnol.* 28, 495-501. 10.1038/nbt.1630 20436461 PMC4840234

[DEV174086C40] Moris N., Pina C. and Arias A. M. (2016). Transition states and cell fate decisions in epigenetic landscapes. *Nature Publishing Group* 17, 693-703. 10.1038/nrg.2016.98 27616569

[DEV174086C41] Murry C. E. and Keller G. (2008). Differentiation of embryonic stem cells to clinically relevant populations: lessons from embryonic development. *Cell* 132, 661-680. 10.1016/j.cell.2008.02.008 18295582

[DEV174086C42] Nakayama R. T., Pulice J. L., Valencia A. M., McBride M. J., McKenzie Z. M., Gillespie M. A., Ku W. L., Teng M., Cui K., Williams R. T. et al. (2017). SMARCB1 is required for widespread BAF complex-mediated activation of enhancers and bivalent promoters. *Nat. Genet.* 49, 1613-1623. 10.1038/ng.3958 28945250 PMC5803080

[DEV174086C43] Nitarska J., Smith J. G., Sherlock W. T., Hillege M. M. G., Nott A., Barshop W. D., Vashisht A. A., Wohlschlegel J. A., Mitter R. and Riccio A. (2016). A functional switch of NuRD chromatin remodeling complex subunits regulates mouse cortical development. *Cell Rep.* 17, 1683-1698. 10.1016/j.celrep.2016.10.022 27806305 PMC5149529

[DEV174086C44] O'Geen H., Echipare L. and Farnham P. J. (2011). Using ChIP-seq technology to generate high-resolution profiles of histone modifications. *Methods Mol. Biol.* 791, 265-286. 10.1007/978-1-61779-316-5_20 21913086 PMC4151291

[DEV174086C45] Perez-Riverol Y., Csordas A., Bai J., Bernal-Llinares M., Hewapathirana S., Kundu D. J., Inuganti A., Griss J., Mayer G., Eisenacher M. et al. (2018). The PRIDE database and related tools and resources in 2019: improving support for quantification data. *Nucleic Acids Res.* 47, D442-D450. 10.1093/nar/gky1106 PMC6323896 30395289

[DEV174086C46] Pierre R. S. and Kadoch C. (2017). Science direct mammalian SWI/SNF complexes in cancer: emerging therapeutic opportunities. *Curr. Opin. Genet. Dev.* 42, 56-67. 10.1016/j.gde.2017.02.004 28391084 PMC5777332

[DEV174086C47] Reyes J. C., Barra J., Muchardt C., Camus A., Babinet C. and Yaniv M. (1998). Altered control of cellular proliferation in the absence of mammalian brahma (SNF2alpha). *EMBO J.* 17, 6979-6991. 10.1093/emboj/17.23.6979 9843504 PMC1171046

[DEV174086C48] Robinson M. D., McCarthy D. J. and Smyth G. K. (2010). edgeR: a Bioconductor package for differential expression analysis of digital gene expression data. *Bioinformatics* 26, 139-140. 10.1093/bioinformatics/btp616 19910308 PMC2796818

[DEV174086C49] Saccone V., Consalvi S., Giordani L., Mozzetta C., Barozzi I., Sandoná M., Ryan T., Rojas-Muñoz A., Madaro L., Fasanaro P. et al. (2014). HDAC-regulated myomiRs control BAF60 variant exchange and direct the functional phenotype of fibro-adipogenic progenitors in dystrophic muscles. *Genes Dev.* 28, 841-857. 10.1101/gad.234468.113 24682306 PMC4003277

[DEV174086C50] Sanjana N. E., Cong L., Zhou Y., Cunniff M. M., Feng G. and Zhang F. (2012). A transcription activator-like effector toolbox for genome engineering. *Nat. Protoc.* 7, 171-192. 10.1038/nprot.2011.431 22222791 PMC3684555

[DEV174086C51] Sen P., Luo J., Hada A., Hailu S. G., Dechassa M. L., Persinger J., Brahma S., Paul S., Ranish J. and Bartholomew B. (2017). Loss of Snf5 induces formation of an aberrant SWI/ SNF complex. *Cell Rep* 18, 2135-2147. 10.1016/j.celrep.2017.02.017 28249160 PMC5424545

[DEV174086C52] Sohn D. H., Lee K. Y., Lee C., Oh J., Chung H., Jeon S. H. and Seong R. H. (2007). SRG3 interacts directly with the major components of the SWI/SNF chromatin remodeling complex and protects them from proteasomal degradation. *J. Biol. Chem.* 282, 10614-10624. 10.1074/jbc.M610563200 17255092

[DEV174086C53] Staahl B. T., Tang J., Wu W., Sun A., Gitler A. D., Yoo A. S. and Crabtree G. R. (2013). Kinetic analysis of npBAF to nBAF switching reveals exchange of SS18 with CREST and integration with neural developmental pathways. *J. Neurosci.* 33, 10348-10361. 10.1523/JNEUROSCI.1258-13.2013 23785148 PMC3685834

[DEV174086C54] Sun X., Hota S. K., Zhou Y.-Q., Novak S., Miguel-Perez D., Christodoulou D., Seidman C. E., Seidman J. G., Gregorio C. C., Henkelman R. M. et al. (2018). Cardiac-enriched BAF chromatin-remodeling complex subunit Baf60c regulates gene expression programs essential for heart development and function. *Biol. Open* 7, bio029512 10.1242/bio.029512 29183906 PMC5829499

[DEV174086C55] Takeuchi J. K. and Bruneau B. G. (2009). Directed transdifferentiation of mouse mesoderm to heart tissue by defined factors. *Nature* 459, 708-711. 10.1038/nature08039 19396158 PMC2728356

[DEV174086C56] Takeuchi J. K., Lou X., Alexander J. M., Sugizaki H., Delgado-Olguín P., Holloway A. K., Mori A. D., Wylie J. N., Munson C., Zhu Y. et al. (2011). Chromatin remodelling complex dosage modulates transcription factor function in heart development. *Nat. Commun.* 2, 187-111. 10.1038/ncomms1187 21304516 PMC3096875

[DEV174086C57] Thomas R., Thomas S., Holloway A. K. and Pollard K. S. (2017). Features that define the best ChIP-seq peak calling algorithms. *Brief. Bioinformatics* 18, 441-450. 10.1093/bib/bbw035 27169896 PMC5429005

[DEV174086C58] van den Boogaard M., Wong L. Y. E., Tessadori F., Bakker M. L., Dreizehnter L. K., Wakker V., Bezzina C. R., t Hoen P. A. C., Bakkers J., Barnett P. et al. (2012). Genetic variation in T-box binding element functionally affects SCN5A/SCN10A enhancer. *J. Clin. Invest.* 122, 2519-2530. 10.1172/JCI62613 22706305 PMC3386824

[DEV174086C59] van den Boogaard M., Smemo S., Burnicka-Turek O., Arnolds D. E., van de Werken H. J. G., Klous P., McKean D., Muehlschlegel J. D., Moosmann J., Toka O. et al. (2014). A common genetic variant within SCN10A modulates cardiac SCN5A expression. *J. Clin. Invest.* 124, 1844-1852. 10.1172/JCI73140 24642470 PMC3973109

[DEV174086C60] Wamstad J. A., Alexander J. M., Truty R. M., Shrikumar A., Li F., Eilertson K. E., Ding H., Wylie J. N., Pico A. R., Capra J. A. et al. (2012). Dynamic and coordinated epigenetic regulation of developmental transitions in the cardiac lineage. *Cell* 151, 206-220. 10.1016/j.cell.2012.07.035 22981692 PMC3462286

[DEV174086C61] Wang W., Xue Y., Zhou S., Kuo A., Cairns B. R. and Crabtree G. R. (1996). Diversity and specialization of mammalian SWI/SNF complexes. *Genes Dev.* 10, 2117-2130. 10.1101/gad.10.17.2117 8804307

[DEV174086C62] Wang Y., Wong R. H. F., Tang T., Hudak C. S., Yang D., Duncan R. E. and Sul H. S. (2013). Phosphorylation and recruitment of BAF60c in chromatin remodeling for lipogenesis in response to insulin. *Mol. Cell* 49, 283-297. 10.1016/j.molcel.2012.10.028 23219531 PMC3786575

[DEV174086C63] Wang X., Lee R. S., Alver B. H., Haswell J. R., Wang S., Mieczkowski J., Drier Y., Gillespie S. M., Archer T. C., Wu J. N. et al. (2017). SMARCB1-mediated SWI/SNF complex function is essential for enhancer regulation. *Nat. Genet.* 49, 289-295. 10.1038/ng.3746 27941797 PMC5285474

[DEV174086C64] Wu J. I., Lessard J., Olave I. A., Qiu Z., Ghosh A., Graef I. A. and Crabtree G. R. (2007). Regulation of dendritic development by neuron-specific chromatin remodeling complexes. *Neuron* 56, 94-108. 10.1016/j.neuron.2007.08.021 17920018

[DEV174086C65] Yang X., Zaurin R., Beato M. and Peterson C. L. (2007). Swi3p controls SWI/SNF assembly and ATP-dependent H2A-H2B displacement. *Nat. Struct. Mol. Biol.* 14, 540-547. 10.1038/nsmb1238 17496903

[DEV174086C66] Yoo A. S., Staahl B. T., Chen L. and Crabtree G. R. (2009). MicroRNA-mediated switching of chromatin-remodelling complexes in neural development. *Nature* 460, 642-646. 10.1038/nature08139 19561591 PMC2921580

[DEV174086C67] Zaidi S., Choi M., Wakimoto H., Ma L., Jiang J., Overton J. D., Romano-Adesman A., Bjornson R. D., Breitbart R. E., Brown K. K. et al. (2013). De novo mutations in histone-modifying genes in congenital heart disease. *Nature* 498, 220-223. 10.1038/nature12141 23665959 PMC3706629

[DEV174086C68] Zambon A. C., Gaj S., Ho I., Hanspers K., Vranizan K., Evelo C. T., Conklin B. R., Pico A. R. and Salomonis N. (2012). GO-Elite: a flexible solution for pathway and ontology over-representation. *Bioinformatics* 28, 2209-2210. 10.1093/bioinformatics/bts366 22743224 PMC3413395

[DEV174086C69] Zhou C. Y., Johnson S. L., Gamarra N. I. and Narlikar G. J. (2016). Mechanisms of ATP-dependent chromatin remodeling motors. *Annu. Rev. Biophys.* 45, 153-181. 10.1146/annurev-biophys-051013-022819 27391925 PMC9157391

