# Supplementary material for: Dynamic BAF chromatin remodeling complex subunit inclusion promotes temporally distinct gene expression programs in cardiogenesis
Source: Development. 2019 Jul 5;146(19):dev174086. doi: 10.1242/dev.174086 (PMC6803373; doi:10.1242/dev.174086)
Supplement: Supplementary information [file develop-146-174086-s1.pdf]

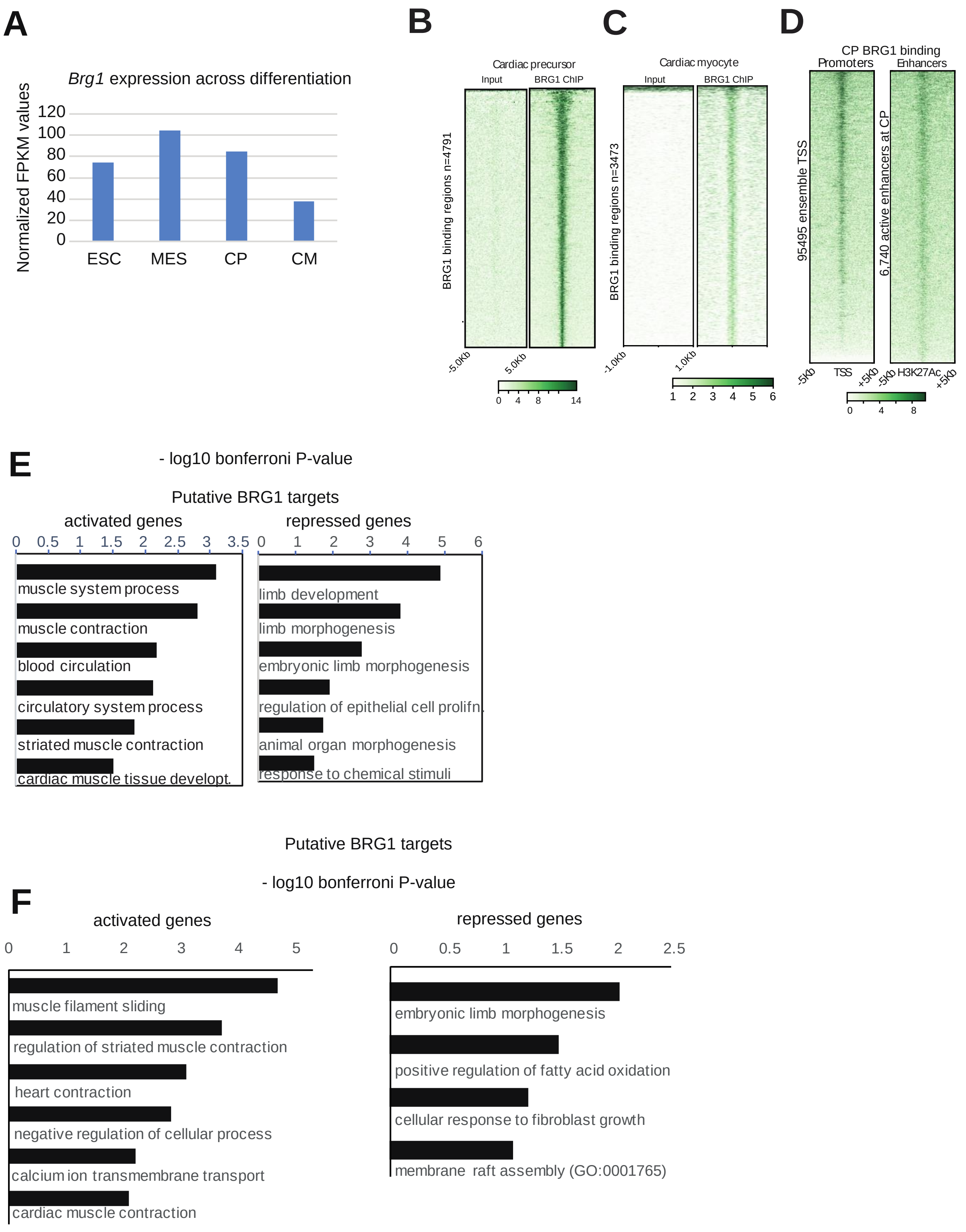

Development • Supplementary information

Fig. S1. *Brg1* expression and BRG1 genomic binding decreases from CP to CM. (*A*) Expression level of *Brg1* mRNA across indicated four different stages of cardiac differentiation. (*B*) Input and BRG1 ChIP signal over 4791 cardiac precursor BRG1 binding sites. (*C*) Input and BRG1 ChIP signal over 3473 cardiac myocyte BRG1 binding sites. (*D*) BRG1 ChIP signal over TSS of all 95495 emsemble genes and 6740 enhancers in cardiac precursors that are enriched for H3K27ac marks. (*E&F*) GREAT analysis of biological processes enriched in two flanking genes within 1Mb (E) or within 100Kb (F) of a BRG1 binding regions at cardiac precursors.

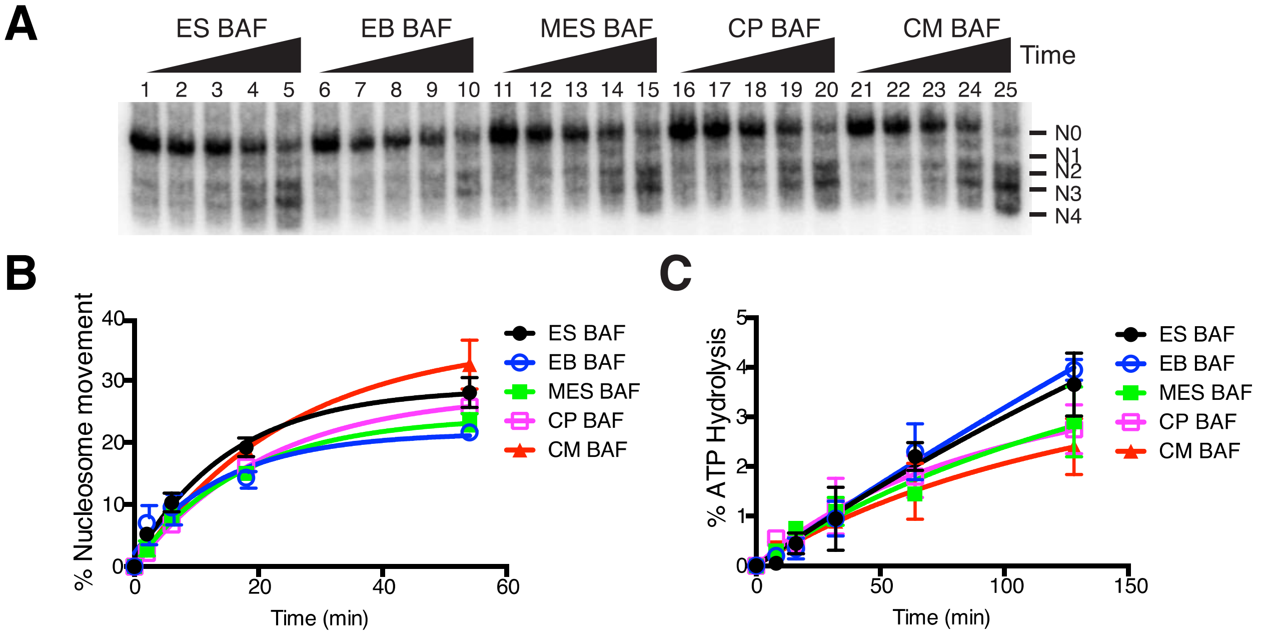

Fig. S2. BRG1 containing complexes from different stages of cardiac differentiation do not significantly change nucleosome repositioning or ATPase activities. (*A*) Nucleosome repositioning by BAF complexes isolated at indicated stages of cardiac differentiation. N0 represents the starting nucleosome position. N1-N4 represent BAF displaced repositioned nucleosomes over time. (*B*) Quantification of nucleosome repositioning. Error bars represent s.e.m of two independent replicates. (*C*) Nucleosome stimulated ATP hydrolysis by BAF complexes isolated at different stages of cardiac differentiation. Error bars represent s.e.m of three independent replicates

Development • Supplementary information

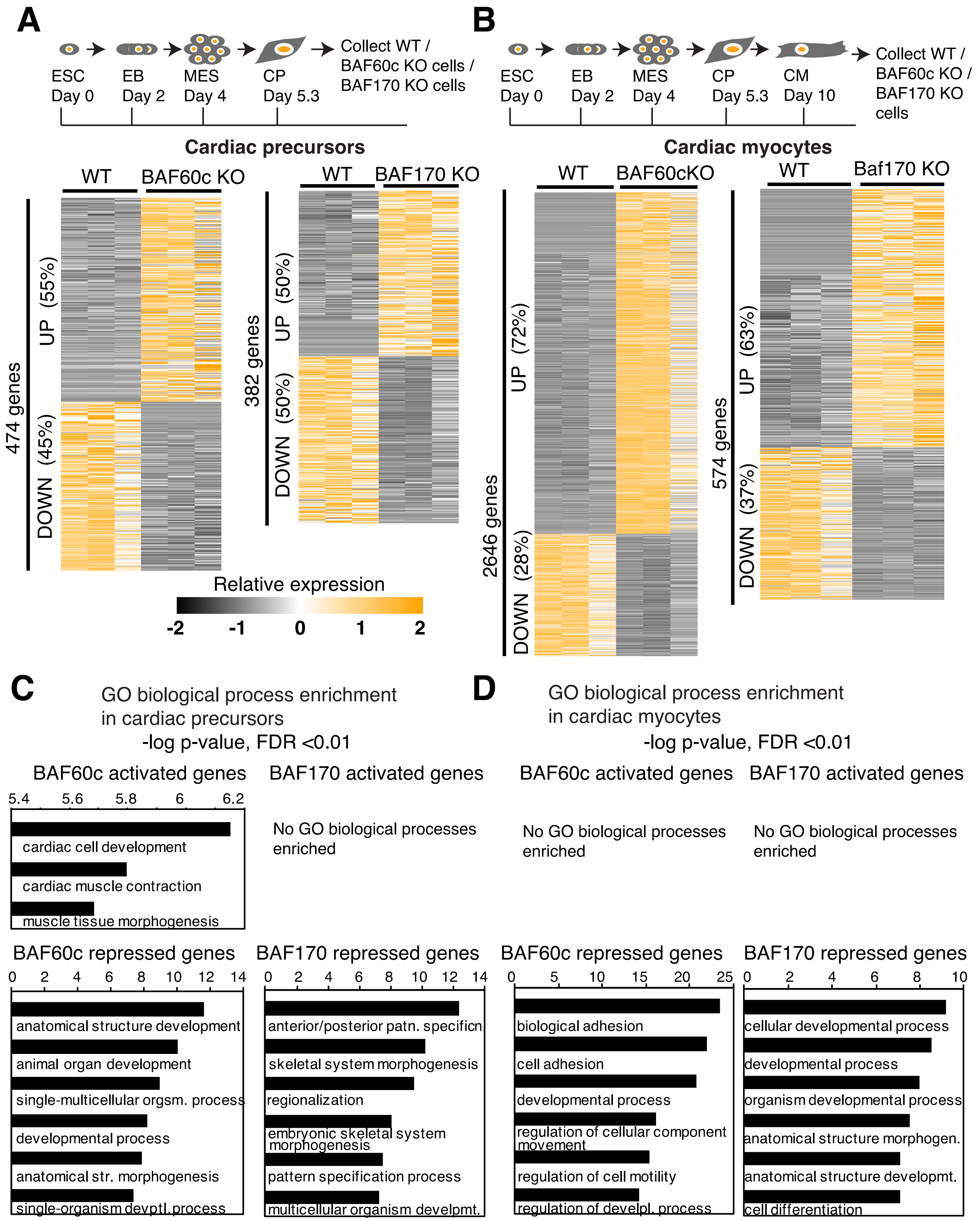

Development • Supplementary information

Fig. S3. RNAseq analysis of BAF60c KO and BAF170 KO in CP and CM stages of cardiac differentiation. (*A*) Schematics of cardiac differentiation and time point of cell collection for RNAseq. Heat map showing genes up or down regulated in absence of BAF60c (left panel) or BAF170 (right panel) in cardiac precursors. (*B*) Same as A but in cardiomyocytes. GO biological processes enriched in *Baf60c* or *Baf170* KO cells in cardiac precursors (*C*) or cardiac myocytes (*D*) are shown. Processes down regulated in mutants were shown on the top panels and processes upregulated shown on bottom panels.

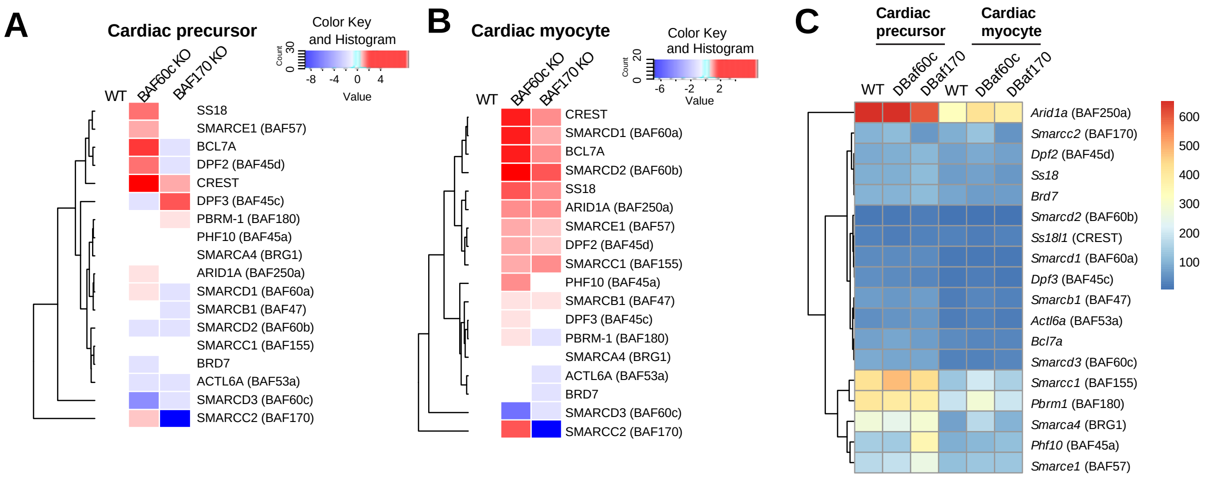

Fig. S4. BRG1 complexes form sub-modules in absence of BAF60c and BAF170

(*A*) Peptide intensities of BRG1 complexes from cardiac precursors lacking BAF60c or BAF170 are normalized to the protein levels of BRG1 and to their WT counterparts.

(*B*) Same as *A*, except in cardiomyocytes. Color bar indicates relative association of proteins with BRG1 with blue, white and red representing depletion (less than 1.25-fold), no change (within 1.25-fold) and enrichment (more than 1.25-fold change) in protein abundance respectively. (*C*) Gene expression analysis of BRG1 associated subunits at the indicated stages in absence of BAF60c or BAF170 were plotted for the indicated genotypes at CP and CM. Color bar shows the normalized median FPKM expression values from three biological replicates.

Development • Supplementary information

Table S1. Peptide intensities of BRG1 associated factors normalized to BRG1, mock and stages of differentiation

Proteins	ES	EB	MES	CP	CM
Arid1a	0.84635404	0.82347087	0.79464565	0.8522635	0.47151328
Bcl7c	0.76068954	0.70875954	0.61892629	0.68690619	0.58030258
Smarce1	0.90827573	0.8944646	0.97562634	0.98481074	1.0921269
Brd7	0.65418882	0.59540354	0.68567642	0.86259994	0.794065
Crabp2	1.48725024	1.54973332	1.64175901	1.38116652	1.14160032
Kpna2	0.66643305	0.65267795	0.55396501	0.48782117	0.21816989
Dpf3	0.07570307	0.07105626	0.32772602	0.47195263	1.21135812
Smarcc1	0.96300145	0.93277923	0.92306897	0.8299736	0.70809903
Pde4d	0.64951504	0.47604558	0.68781801	0.32729406	0.07860122
Smarca4	1	1	1	1	1
Brd9	0.64693832	0.51006836	0.30364949	0.46750067	0.44064719
Dpf2	0.91781479	0.91065905	0.90655377	0.90015406	0.73071707
Smarcd1	1.0855543	1.03001925	0.94010895	0.84909846	0.62104265
Ss18	0.91055028	0.89738371	0.90840276	0.89533764	0.81809907
Smarcd3	0.12094294	0.07903315	0.03115146	0.79246978	1.08007784
Smarcc2	0.47587393	0.47054134	0.58260018	0.99207892	1.17529351
Cc2d1b	1.33864929	1.41636558	1.37560897	1.30367742	2.10001608
Pbrm1	0.74688856	0.69929075	0.76961507	0.93665106	0.80779027
Ss18l1	0.60162015	0.50924822	0.43455561	0.68032909	0.85274756
Gltscr1l	0.66621073	0.47818469	0.15722018	0.26518071	0.15897126
Bcl7b	1.12468805	1.05521077	0.94866147	0.92193426	0.78693866
Smarcd2	0.68122717	0.64463114	0.75189112	0.84359646	0.33821632
Actl6b	0.8168805	0.54937634	0.75029697	0.70971716	0.68228738
Phf10	0.79514986	0.75998542	0.81354966	0.94533506	0.9103153
Dpf1	0.68951125	0.59635924	0.3287721	0.42801159	0.00716695
Smarcb1	0.76032473	0.7176474	0.72672477	0.85765972	0.82337439
Actl6a	0.86368048	0.83509459	0.88661422	0.97995164	0.98916593
Bcl7a	0.36640499	0.31964959	0.48074457	0.64705114	0.55850029
Wdr5	0.45543024	0.30685646	0.33280484	0.63190965	0.94279359
Cse1l	0.19710033	0.17531935	0.03214023	0.44486471	0.40938411
Arid2	0.24333226	0.2099816	0.2856994	0.41289599	0.29387379
Arid1b	0.04174682	0.04295445	0.07626774	0.07449969	0.05688183

Development • Supplementary information

Table S2. Raw data of BRG1, BAF170, BAF60c and mock immunopuriifcation associated proteins

Click here to Download Table S2

Table S3. MS data normalization for BRG1 associated proteins

Click here to Download Table S3

Table S4. Significant *Brg1* regulated genes in cardiac precursors and cardiac myocytes

Click here to Download Table S4

Table S5. Significant *Baf60c* regulated genes in cardiac precursors and cardiac myocytes

Click here to Download Table S5

Table S6. Significant *Baf170* regulated genes in cardiac precursors and cardiac myocytes

Click here to Download Table S6
